# Sex differences in the awareness of emergency contraceptive pills associated with unmarried Korean university students’ intention to use contraceptive methods: an online survey

**DOI:** 10.1186/s12978-015-0076-x

**Published:** 2015-09-22

**Authors:** Hae Won KIM

**Affiliations:** The research institute of nursing science, College of Nursing, Seoul National University, Taehakro 103, Jongro-Gu, Seoul South Korea 110-799

## Abstract

**Background:**

Awareness of emergency contraceptive pills (ECP) associated with an intention to use other contraceptive methods has rarely been investigated. This study compared the ECP awareness of males and females and its associations with intention to use four other contraceptive methods (condoms, oral contraceptive pills, and withdrawal and rhythm methods) in unmarried university students in Korea. This study explores the importance of ECP awareness in university students’ contraceptive education.

**Methods:**

A descriptive cross-sectional study design was employed, in which 1372 unmarried university students (men, *n* = 755, women, *n* = 617) answered a Web-based survey. Sex differences in ECP awareness and four contraceptive intentions, and associations between ECP awareness and contraceptive intentions between sex were analysed using independent *t*-tests and *χ*^2^ test. Variables yielding significant associations with contraceptive intentions (*p* < 0.05) were included in a logistic regression using the adjusted odds ratio (AOR) to estimate the impact of ECP awareness on students’ contraceptive intentions.

**Results:**

Awareness of ECP was found in 88.2 % of participants, which was generally positive. There were significant sex differences in some ECP awareness and students’ contraceptive intentions, and in the associations between previous ECP use and ECP awareness between male and female university students. In men, the belief that “ECP can cause sex with multiple partners” was associated with intention to use the rhythm method (AOR = 1.61, 95 % confidence interval [CI] = 1.02–2.56). For women, the belief that “ECP is necessary in case of condom breakage” was associated with intention to use the withdrawal (AOR = 058, 95 % CI = 0.37–0.93) or rhythm (AOR = 0.36, 95 % CI = 0.16–0.84) methods, and “ECP should be prescribed by a doctor” was associated with the intention to use the rhythm method (AOR = 0.45, 95 % CI = 0.26–0.77).

**Conclusions:**

ECP awareness was associated with the intentions of students to use withdrawal or rhythm methods. The sex-specific approach in the examination of students’ contraceptive intentions and their determinants was helpful.

## Background

There are increasing trends of premarital sex, unwanted pregnancy, and abortion with a lack of successful contraception among university students in Korea [1, 2]. These university students are considered to be vulnerable to sexual health problems especially at this stage of their life; therefore, the importance of contraception for them is highlighted [1]. Researchers have focused on the need to better promote emergency contraceptive pills (ECP) to university students worldwide [3–5] because it is known as being a safe and effective solution for students who might face abortion without the timely use of ECP [6]. ECP has been considered as a sensible choice to prevent unwanted pregnancy 73 among sexually active women aged 16 to 46 years living in five European countries [7], but it 74 was also reported that increasing the use of ECP in the United Kingdom did not reduce the 75 pregnancy rate [8].

The use of ECP in Korea has been approved by the Korean Ministry of Health since 2001. Currently ECP, including the Yuzpe regimen and levonorgestrel, are available, but these should be prescribed by a doctor and then purchased at a pharmacy [9]. The rate of ECP use by Korean university students aged 17 to 30 years was found to be 13.2 % in a survey reported in 2008 [2], but the reported rate of ECP use was only 0.9 % in Korean middle- and high-school students [10]. This means that ECP were used more by Korean university students than by adolescents, but it also appears that ECP cannot be considered as a convenient method of contraception for Korean university students. Meanwhile, four types of contraceptive methods were found to be most frequently used by Korean university students: condoms, the withdrawal method, oral contraceptive pills, and the rhythm method [11]. To date, little research has been performed on ECP awareness in Korean university students. Previous studies have indicated that these students perceive ECP as being a favorable method, with them generally having positive attitudes toward using ECP [2, 12]. One study found that the students’ positive attitudes toward ECP use was associated with higher intentions to use a condom [2], but the Korean students’ ECP awareness in relation to their intention to use other contraceptive methods was not explored.

It was assumed that ECP awareness could be extended to the choice of other contraceptive methods because then ECP awareness could be associated with intention to use other contraceptive methods as a proxy for real-world choices of contraceptive methods. This is in line with Ajen’s postulation that attitudes could influence intentions, and subsequently the intention predicts real-world behaviour [13]. Although the accessibility to ECPs is limited for Korean university students, their ECP awareness and its effects on intentions and contraceptive choices should be explored. Subsequently, it will be possible to confirm the expanded role of ECP awareness in Korean university students’ contraception use or intentions, which would be helpful in planning contraceptive education for these students. A sex-specific examination should be considered to identify the associations between ECP awareness and contraceptive intentions because the studies have confirmed sex differences in ECP awareness [2, 4, 5], and the effect of men on communicating contraceptive choices were significant [11, 14–16].

This study aimed to compare ECP awareness in males and females and its associations with contraceptive intentions, including condom use, oral contraceptive pills, and the withdrawal and rhythm methods, which are the most popularly used by Korean university students. This study also evaluated the influence of previous ECP use on current ECP awareness according to sex.

## Methods

### Subjects and data collection procedures

The Seoul National University Institutional Review Board approved the research protocol. Undergraduate and graduate students enrolled at the Seoul National University between November 2013 and April 2014 received an email about this study. After approval, students were emailed once a month for 5 months with the support of the administrative office of the university. Students who willingly participated in this study directly accessed the online survey Website at http://research.joongang.com/survey.php?v = y&id = 13-9-1291. Subjects were included in the study if they were unmarried and were undergraduate or graduate students currently attending Seoul National University. Subjects were requested to read the study protocols and to complete an informed consent form. No financial incentive was offered and subjects were informed that they could contact the research team by email or telephone if they needed counselling or to discuss private information. Data were collected between November 15, 2013, and April 30, 2014 using a Web-based survey. All subjects were anonymously coded upon entry to the survey. If the subjects completed the survey once, the online survey control program did not allow them to respond to the same survey.

A total of 1449 students completed the survey, which constituted 5.18 % of the 27,967 students at Seoul National University in April 2013 (16,712 undergraduate students and 11,255 graduate students). Seventy-two respondents were married, so they were excluded; therefore, the study analysis was based on a final sample of 1372 subjects.

### Measures

The contents and constructs of the questionnaire were validated by two contraceptive research experts and tested among ten university students aged 22–24 years prior to the survey. The survey identified the subjects’ ECP awareness, intentions to use contraceptive methods, and demographic and sexual history characteristics.

#### ECP awareness

First, subjects were asked whether they have heard of ECP, then they were asked to agree or disagree with the following questions to extract further details of their ECP awareness:

1. ECP use is necessary.
2. ECP should be available as an over-the-counter drug.
3. ECP should be prescribed by a doctor.
4. ECP is necessary for women’s health.
5. ECP is necessary in cases of rape, condom breakage, and unwanted sex.
6. ECP will reduce unwanted pregnancy.
7. ECP can cause promiscuity.

With respect to whether the subjects knew the maximum time for taking an ECP after unwanted sex, answers were given in six categories (within 12, 24, 48, 72, 120 h after unwanted sex, and don’t know).

#### Intentions to use contraceptive methods

Contraceptive intentions were assumed to be planned prior to sex in this study; therefore, the intention to use ECP was not measured because ECP was considered to be the secondary method, not the primary method when no contraceptive method was planned. The students were informed that their contraceptive intentions in this study were confined to the prevention of an unwanted pregnancy, even though there was the complementary intention to prevent sexually transmitted infections (STIs).

The four types of contraceptive methods (condoms, oral contraceptive pills, and the withdrawal and rhythm methods) were chosen to measure contraceptive intentions for this study because those were the most popular contraceptive methods in the young and the general Korean population [1, 11, 16]. Therefore, other methods such as an intrauterine device (long-acting contraceptive) or non-oral hormonal types of contraception were excluded from this study. Students alternated or combined contraceptive choices to increase the success of the contraception [17, 18]. Consistency, regularity, and voluntariness were the critical attributes in the evaluation of the students’ contraceptive intentions [14, 17]. Based on these attributes, the subjects were asked to respond to all the possible intentions of using these four contraceptive methods with three items as follows:

1. I will choose this method myself.
2. I will use this method consistently.
3. I will choose this method without another’s recommendation.

Prior to this survey, the item validity was confirmed by two experts using a 5-point Likert scale (1 = not necessary at all; 5 = essential); finally, all three items were confirmed as being essential (5). Each intention was assessed on a 5-point Likert scale (1 = not at all; 5 = very much); therefore, the possible range for each intention score was 3 to 15. Higher scores for each method indicated that students had greater intentions to use that method. When answering their intentions, subjects were asked to imagine that their decision could be made in agreement with their actual or imaginary sexual partners.

#### Demographic and sexual history characteristics

As a baseline, details of the age, grades, study major, religion, and levels of smoking and alcohol consumption from each respondent were collected. The students’ levels of sexual experience, number of sexual partners if they were sexually active, sexuality patterns, STI history, previous contraceptive use (ECP, condoms, oral contraceptive pills, or withdrawal or rhythm methods), and previous experiences (unwanted sex, unwanted pregnancy, or abortion) were included.

### Statistical analysis

Descriptive statistics were analysed, including frequencies, means, proportions, standard deviations, and percentages. Sex differences in demographic and sexual characteristics, ECP awareness, and contraceptive intentions were analysed using the *χ*^2^ test for homogeneity of nominal variables, and independent *t*-tests for continuous variables. Potential associations between independent variables (ECP awareness, demographic, and sexual history characteristics) and dependent variables (intention to use one of four contraceptive methods) were analysed using the *χ*^2^ test.

Among the independent variables, the following data were converted into dichotomous scales according to the median: age was scored as 0 for “18–23 years” in men, “18–22 years” in women, and 1 for “24–40 years” in men, “23–41 years” in women. The number of sexual partners was scored as 0 and 1 for “one” and “multiple”, respectively. Smoking levels were scored as 0 and 1 for “never experienced” and “ever smoked”, respectively. Alcohol consumption was coded as 0 and 1 for “less than once a week” and “more than once a week”, respectively. In students’ ECP awareness, the maximum time for taking ECP was scored as 0 for “incorrect” or “don’t know” and 1 for “correct”. Details of other ECP awareness were coded as 0 and 1 for “disagree” and “agree”, respectively. Other independent variables (religion, STI history, previous experience with contraceptive methods, unwanted pregnancy, and abortion) were scored as 0 and 1 for “no” and “yes” responses.

The mean scores of the dependent variables were converted into dichotomous scales according to “low”/“high” intentions to use a contraceptive method: condoms (3–12/13–15 in men and women); oral contraceptive pills (3–6/7–15 in men, 3–7/8–15 in women), withdrawal method (3–5/6–15 in men and women), and rhythm method (3–6/7–15 in men and women). Values for the intentions were assigned, with 0 indicating “low intention” and 1 indicating “high intention”.

To identify the impact of ECP awareness on students’ contraceptive intentions, variables yielding significant associations at *p* < 0.05 were included in a logistic regression to calculate adjusted odds ratios (AORs) and 95 % confidence intervals (CIs) between the dependent variables and independent variables at *p* < 0.05. SPSS Statistics software (v. 21.0; IBM SPSS, Armonk, NY, USA) was used for all statistical analyses.

## Results

### Study sample characteristics

The mean ages of the students were 24.04 ± 3.65 years and 22.94 ± 2.62 years (mean ± standard deviation; range 13–23 years, 18–41 years), in men and women, respectively. Men comprised 55.03 % (*n* = 755) and women 44.97 % (*n* = 617) of the 1372 subjects. The distribution of students’ majors indicated that 38.7 % (*n* = 531) were in science and engineering, 19.2 % (*n* = 264) human science and social science, 11.7 % (*n* = 161) agriculture and biotechnology, 8.2 % (*n* = 113) medicine and nursing, and 7.9 % (*n* = 108) education. The participant’s distribution by grade were compared with actual student numbers at the university; the first year students were 12.8 % (*n* = 176; actual, 13.4 %), the second years were 12.2 % (*n* = 168; actual, 13.1 %), the third years were 13.6 % (*n* = 186; actual, 12.9 %), the fourth years were 20.5 % (*n* = 281; actual, 20.3 %), and the graduate students were 40.9 % (*n* = 561; actual, 40.2 %). The results appeared to represent a balanced proportion of students across their grades and were reflective of the diverse majors. Analysis of the homogeneity of demographic and sex characteristics indicated that there were significant sex differences in age (*χ*^2^ = 26.84, *p <* 0.001), grade (*χ*^2^ = 32.63, *p <* 0.001), major (*χ*^2^ = 196.90, *p <* 0.001), religion (*χ*^2^ = 5.84, *p =* 0.02), smoking (*χ*^2^ = 44.0, *p <* 0.001), alcohol drinking (*χ*^2^ = 33.99, *p <* 0.001), sexual experience (*χ*^2^ = 25.78, *p <* 0.001), number of sexual partners (*χ*^2^ = 14.08, *p <* 0.001), sexuality patterns (*χ*^2^ = 17.66, *p =* 0.001), STI history (*χ*^2^ = 17.25, *p <* 0.001), previous contraceptive use (ECP [*χ*^2^ = 29.74, *p <* 0.001], condoms [*χ*^2^ = 5.22, *p =* 0.03], oral contraceptive pills [*χ*^2^ = 21.51, *p <* 0.001], and rhythm method (*χ*^2^ = 4.26, *p =* 0.04]), and previous unwanted sex experience (*χ*^2^ = 26.99, *p <* 0.001) (Table 1).

**Table 1:** Demographic and sexual history characteristics by sex (*n =* 1372)

Characteristic	Category	Total	Men (*n =* 755)	Women (*n =* 617)	*χ* ^2^ (*p*) or *t* (*p*)
			*n* (%) or mean ± SD		
Demographic characteristics					
Age	<22 years	764 (55.7)	373 (49.4)	391 (63.4)	26.84 (<0.001)
	>22 years	608 (44.3)	382 (50.6)	226 (36.6)	
		23.54 ± 3.60	24.04 ± 3.65	22.94 ± 2.62	32.63 (<0.001)
Grade	Freshman	176 (12.8)	78 (10.3)	98 (15.9)	20.42 (<0.001)
	Sophomore	168 (12.2)	104 (13.8)	64 (10.4)	
	Junior	186 (13.6)	105 (13.9)	81 (13.1)	
	Senior	281 (20.5)	136 (18.0)	145 (23.5)	
	Graduate student	561 (40.9)	332 (44.0)	229 (37.1)	
Major	Humanities and society	264 (19.2)	118 (15.6)	146 (23.7)	196.90 (<0.001)
	Business administration	34 (2.5)	21 (2.8)	13 (2.1)	
	Science and engineering	531 (38.7)	404 (53.5)	127 (20.6)	
	Agriculture biotechnology	161 (11.7)	66 (8.7)	95 (15.4)	
	Medicine and nursing	113 (8.2)	33 (4.4)	80 (13.0)	
	Law	14 (1.0)	9 (1.2)	5 (0.8)	
	Education	108 (7.9)	54 (7.2)	54 (8.8)	
	Veterinary medicine and pharmacy	57 (4.2)	27 (3.6)	30 (4.9)	
	Music and art	67 (4.9)	10 (1.3)	57 (9.2)	
	Free or associated major	23 (1.7)	13 (1.7)	10 (1.6)	
Religion	No	519 (37.8)	264 (35.0)	255 (41.3)	5.84 (0.02)
	Yes	853 (62.2)	491 (65.0)	362 (58.7)	
Smoking	Non-smoking	1067 (77.8)	538 (71.3)	529 (85.7)	44.00 (<0.001)
	Previous smoking	176 (12.8)	118 (15.6)	58 (9.4)	
	Currently smoking	129 (9.4)	99 (13.1)	30 (4.9)	
Alcohol drinking	None	102 (7.4)	44 (5.8)	58 (9.4)	33.99 (<0.001)
	1–2/month	746 (54.4)	374 (49.5)	372 (60.3)	
	1/week	367 (26.7)	233 (30.9)	134 (21.7)	
	2–3/week	143 (10.4)	92 (12.2)	51 (8.3)	
	over	14 (1.0)	12 (1.6)	2 (0.3)	
Sexual history characteristics					
Sexual experience	Never experienced	507 (37.0)	248 (32.8)	259 (42.0)	25.78 (<0.001)
	Experienced with different partners	200 (14.6)	140 (18.5)	60 (9.7)	
	Experienced with fixed partner	665 (48.5)	367 (48.6)	298 (48.3)	
Number of sexual partners (*n =* 865)		3.48 ± 5.71	4.09 ± 7.04	2.62 ± 2.69	14.08 (<0.001)
Sexuality patterns (*n =* 865)	Opposite sex	839 (97.0)	490 (96.6)	349 (97.5)	17.66 (0.001)
	Same sex	11 (1.3)	10 (2.0)	1 (0.3)	
	Both	15 (1.7)	7 (1.4)	8 (2.2)	
STI experiences (*n =* 865)	No	830 (96.0)	493 (97.2)	337 (94.1)	17.25 (<0.001)
	Yes	35 (4.0)	14 (2.8)	21 (5.9)	
Previous use of emergency contraceptive pills (*n =* 865)	No	682 (78.8)	432 (85.2)	250 (69.8)	29.74 (<0.001)
	Yes	183 (21.2)	75 (14.8)	108 (30.2)	
Previous condom use (*n =* 865)	No	33 (3.8)	13 (2.6)	20 (5.6)	5.22 (0.03)
	Yes	832 (96.2)	494 (97.4)	338 (94.4)	
Previous use of oral contraceptive pills (*n =* 865)	No	693 (80.1)	433 (85.4)	260 (72.6)	21.51 (<0.001)
	Yes	172 (19.9)	74 (14.6)	98 (27.4)	
Previous use of withdrawal method (*n =* 865)	No	629 (72.7)	372 (73.4)	257 (71.8)	0.27 (0.64)
	Yes	236 (27.3)	135 (26.6)	101 (28.2)	
Previous use of rhythm method (*n =* 865)	No	521 (60.2)	320 (63.1)	201 (56.1)	4.26 (0.04)
	Yes	344 (39.8)	187 (36.9)	157 (43.9)	
Previous unwanted sex (*n =* 865)	No	762 (88.1)	471 (92.9)	291 (81.3)	26.99 (<0.001)
	Yes	103 (11.9)	36 (7.1)	67 (18.7)	
Previous unwanted pregnancy (*n =* 865)	No	822 (95.0)	483 (95.3)	339 (94.7)	0.15 (0.75)
	Yes	43 (5.0)	24 (4.7)	19 (5.3)	
Previous abortion (*n =* 865)	No	836 (96.6)	494 (97.4)	342 (95.5)	2.35 (0.13)
	Yes	29 (3.4)	13 (2.6)	16 (4.5)	

*STI* sexually transmitted infection

### Sex differences in ECP awareness and intention to use four contraceptive methods

ECP awareness was found in 88.2 % of students, 98.3 % showed agreement on the use of ECP, and 99.7 % agreed with the use of ECP in the case of rape. Students who were less supportive of the use of ECP were those who felt that ECP use resulted in sex with multiple partners (31.7 %), that doctors should prescribe ECP (39.4 %), and that ECP should be an over-the-counter drug (56.8 %). In other areas of ECP awareness, subjects showed that they were relatively positive about the uses of ECP in cases of condom breakage, and unwanted sex, women’s health, and reducing unwanted pregnancy. However, only 35 % of the respondents identified the maximum time for taking ECP as within 72 h correctly.

There were significant sex differences in ECP awareness between male and female students: the use of ECP (*χ*^2^ = 3.97, *p =* 0.05), ECP should be prescribed by a doctor (*χ*^2^ = 4.42, *p =* 0.04), and ECP is necessary for women’s health (*χ*^2^ = 4.12, *p =* 0.04). In regard to contraceptive intentions, there were significant sex differences between male and female students in condom use (*t* = 4.73, *p =* 0.03), oral contraceptive pills (*t* = 16.12, *p <* 0.001), and withdrawal method (*t* = 5.73, *p =* 0.02) (Table 2).

**Table 2:** Awareness of emergency contraceptive pills and intentions to use the four contraceptive methods by sex (*n =* 1372)

Characteristic	Category	Total	Men (*n =* 755)	Women (*n =* 617)	*χ* ^2^ (*p*) or *t* (*p*)
			*n* (%) or mean ± SD		
ECP awareness					
Have you ever heard about ECP?	No	162 (11.8)	101 (13.4)	61 (9.9)	3.97 (0.05)
	Yes	1210 (88.2)	654 (86.6)	556 (90.1)	
ECP use is necessary (*n =* 1210)	Disagree	20 (1.7)	15 (2.3)	5 (0.9)	3.59 (0.07)
	Agree	1190 (98.3)	639 (97.7)	551 (99.1)	
ECP should be available OTC (*n =* 1210)	Disagree	487 (40.2)	254 (38.8)	233 (41.9)	1.18 (0.29)
	Agree	723 (56.8)	400 (61.2)	323 (58.1)	
ECP should be prescribed by a doctor (*n =* 1210)	Disagree	733 (60.6)	414 (63.3)	319 (57.4)	4.42 (0.04)
	Agree	477 (39.4)	240 (36.7)	237 (42.6)	
ECP is necessary for women’s health (*n =* 1210)	Disagree	244 (20.2)	146 (22.3)	98 (17.6)	4.12 (0.04)
	Agree	966 (79.8)	508 (77.7)	458 (82.4)	
ECP is necessary in cases of					
rape (*n =* 1210)	Disagree	4 (0.3)	2 (0.3)	2 (0.4)	0.03 (1.00)
	Agree	1206 (99.7)	652 (99.7)	554 (99.6)	
of condom breakage (*n =* 1210)	Disagree	179 (14.8)	88 (13.5)	91 (16.4)	2.02 (0.17)
	Agree	1031 (85.2)	566 (86.5)	465 (83.6)	
unwanted sex (*n =* 1210)	Disagree	101 (8.3)	62 (9.5)	39 (7.0)	2.39 (0.14)
	Agree	1109 (91.7)	592 (90.5)	517 (93.0)	
ECP will reduce unwanted pregnancy (*n =* 1210)	Disagree	99 (8.2)	59 (9.0)	40 (7.2)	1.34 (0.29)
	Agree	1111 (91.8)	595 (91.0)	516 (92.8)	
ECP can cause sex with multiple partners (*n =* 1210)	Disagree	826 (68.3)	458 (70.0)	368 (66.2)	2.05 (0.16)
	Agree	384 (31.7)	196 (30.0)	188 (33.8)	
Maximum time for taking ECP (*n =* 1210)	Within 12 h	84 (6.9)	52 (8.0)	32 (5.8)	7.61 (0.18)
	Within 24 h	267 (22.1)	145 (22.2)	122 (21.9)	
	Within 48 h	343 (28.3)	182 (27.8)	161 (29.0)	
	Within 72 h	424 (35.0)	220 (33.6)	204 (36.7)	
	Within 120 h	5 (0.4)	1 (0.2)	4 (0.7)	
	I don’t know	87 (7.2)	54 (8.3)	33 (5.9)	
Intentions to use contraceptive methods (*n =* 1372)					
Condom		12.22 ± 2.54	12.36 ± 2.54	12.06 ± 2.53	4.74 (0.03)
Oral contraceptive pill		6.99 ± 2.95	6.70 ± 2.76	7.34 ± 3.14	16.12 (<0.001)
Withdrawal method		5.60 ± 2.88	5.43 ± 2.87	5.80 ± 2.87	5.73 (0.02)
Rhythm method		6.71 ± 3.39	6.56 ± 3.23	6.90 ± 3.56	3.49 (0.06)

*OTC* over the counter; *ECP* emergency contraceptive pills

### ECP awareness and intention to use four contraceptive methods according to previous ECP use

Table 3 lists the results for the sex similarities in ECP awareness among previous ECP users. Students who were experienced in ECP using were more knowledgeable about the maximum time for taking ECP (*χ*^2^ = 10.01 and *p <* 0.04, and *χ*^2^ = 20.54 and *p* < 0.001 in male (having ECP users as sexual partners) and female students, respectively, and they agreed that ECP should be used in cases of condom breakage (*χ*^2^ = 4.64, *p =* 0.03; *χ*^2^ = 7.86, *p <* 0.01) and that ECP reduces unwanted pregnancy (*χ*^2^ = 4.06, *p =* 0.05; *χ*^2^ = 7.89, *p <* 0.01), relative to inexperienced males (having ECP nonusers as sexual partners) and female students. However, male students with sexual partners who were experienced in using ECP had more positive opinions about ECP-related women’s health (*χ*^2^ = 6.38, *p =* 0.01) than did students with sexual partners who were inexperienced in using ECP.

**Table 3:** Sex differences in ECP awareness by levels of previous ECP use (*n =* 1210)

Characteristic	Category	Total	Men (*n* = 654)		*χ* ^2^ (*p*) or *t* (*p*)	Total	Women (*n =* 556)		*χ* ^2^ (*p*) or *t* (*p*)
			Unused ECP^a^ (*n =* 580)	Used ECP^b^ (*n =* 580)			Unused ECP (*n =* 448)	Used ECP (*n =* 108)	
			*n* (%) or mean ± SD				*n* (%) or mean ± SD		
Maximum time for taking ECP	Incorrect	434 (66.4)	397 (68.4)	37 (50.0)	10.01 (<0.01)	352 (63.3)	304 (67.9)	48 (44.4)	20.54 (<0.001)
	Correct	220 (33.6)	183 (31.6)	37 (50.0)		204 (36.7)	144 (32.1)	60 (55.6)	
ECP use is necessary	Disagree	15 (2.3)	14 (2.4)	1 (1.4)	0.33 (1.00)	5 (0.9)	3 (0.7)	2 (1.9)	1.37 (0.25)
	Agree	639 (97.7)	566 (97.6)	73 (98.6)		551 (99.1)	445 (99.3)	106 (98.1)	
ECP should be available OTC	Disagree	254 (38.8)	228 (39.3)	26 (35.1)	0.48 (0.53)	233 (41.9)	186 (41.5)	47 (43.5)	0.14 (0.75)
	Agree	400 (61.2)	352 (60.7)	48 (64.9)		323 (58.1)	262 (58.5)	61 (56.5)	
ECP should be prescribed by a doctor	Unnecessary	414 (63.3)	371 (64.0)	43 (58.1)	0.97 (0.37)	319 (57.4)	257 (57.4)	62 (57.4)	0.00 (1.00)
	Necessary	240 (36.7)	209 (36.0)	31 (41.9)		237 (42.6)	191 (42.6)	46 (42.6)	
ECP is necessary for women’s health	No	146 (22.3)	138 (23.8)	8 (10.8)	6.38 (0.01)	98 (17.6)	85 (19.0)	13 (12.0)	2.88 (0.09)
	Yes	508 (77.7)	442 (76.2)	66 (89.2)		458 (82.4)	363 (81.0)	95 (88.0)	
ECP is necessary in cases of									
Rape	No	2 (0.3)	2 (0.3)	0 (0.0)	0.26 (1.00)	2 (0.4)	2 (0.4)	0 (0.0)	0.48 (1.00)
	Yes	652 (99.7)	578 (99.7)	74 (100.0)		554 (99.6)	446 (99.6)	108 (100.0)	
Condom breakage	No	88 (13.5)	84 (14.5)	4 (5.4)	4.64 (0.03)	91 (16.4)	83 (18.5)	8 (7.4)	7.86 (<0.01)
	Yes	566 (86.5)	496 (85.5)	70 (94.6)		465 (83.6)	365 (81.5)	100 (92.6)	
Unwanted sex	No	62 (9.5)	57 (9.8)	5 (6.8)	0.72 (0.53)	39 (7.0)	33 (7.4)	6 (5.6)	0.44 (0.68)
	Yes	592 (90.5)	523 (90.2)	69 (93.2)		517 (93.0)	415 (92.6)	102 (94.4)	
ECP will reduce unwanted Pregnancy	No	59 (9.0)	57 (9.8)	2 (2.7)	4.06 (0.05)	40 (7.2)	39 (8.7)	1 (0.9)	7.89 (<0.01)
	Yes	595 (91.0)	523 (90.2)	72 (97.3)		516 (92.8)	409 (91.3)	107 (99.1)	
ECP can cause sex with multiple partners	No	458 (70.0)	405 (69.8)	53 (71.6)	0.10 (0.79)	368 (66.2)	293 (65.4)	75 (69.4)	0.64 (0.50)
	Yes	196 (30.0)	175 (30.2)	21 (28.4)		188 (33.8)	155 (34.6)	33 (30.6)	

*OTC* over the counter; *ECP* emergency contraceptive pills

^a^Men with sexual partners who were inexperienced in ECP; ^b^Men with sexual partners who were experienced in ECP

### Sex differences in factors associated with intention to use four contraceptive methods

Table 4 illustrates the results for the sex-related associations between demographic, sexual history characteristics, ECP awareness, and intention to use four contraceptive methods.

**Table 4:** Associations between demographic and sex characteristics and ECP awareness, and the four intentions to use contraceptives by sex

Characteristic	Category	Intention to use condoms			Intention to use oral contraceptive pills			Intention to use withdrawal method			Intention to use rhythm method		
		Low *n* (%)	High *n* (%)	*χ* ^2^ (*p*) or *t* (*p*)	Low *n* (%)	High *n* (%)	*χ* ^2^ (*p*) or *t* (*p*)	Low *n* (%)	High *n* (%)	*χ* ^2^ (*p*) or *t* (*p*)	Low *n* (%)	High *n* (%)	*χ* ^2^ (*p*) or* t* (*p*)
Men (*n =* 654)													
Age	18–23	203 (52.9)	116 (43.0)	6.22 (0.01)	189 (49.3)	130 (48.0)	0.12 (0.75)	175 (50.0)	144 (47.4)	0.45 (0.53)	195 (48.5)	124 (49.2)	0.03 (0.87)
	24–40	181 (47.1)	154 (57.0)		194 (50.7)	141 (52.0)		175 (50.0)	160 (52.6)		207 (51.5)	128 (50.8)	
Religion	No	137 (35.7)	86 (31.9)	1.03 (0.32)	129 (33.7)	94 (34.7)	0.07 (0.80)	111 (31.7)	112 (36.8)	1.90 (0.19)	122 (30.3)	101 (40.1)	6.53 (0.01)
	Yes	247 (64.3)	184 (68.1)		254 (66.3)	177 (65.3)		239 (68.3)	192 (63.2)		280 (69.7)	151 (59.9)	
Smoking	Never experienced	328 (85.4)	233 (86.3)	0.10 (0.82)	323 (84.3)	238 (87.8)	1.58 (0.21)	199 (85.4)	262 (86.2)	0.08 (0.82)	350 (87.1)	211 (83.7)	1.41 (0.25)
	Smoking	56 (14.6)	37 (13.7)		60 (15.7)	33 (12.2)		51 (14.6)	42 (13.8)		52 (12.9)	41 (16.3)	
Alcohol drinking	Rare	222 (57.8)	147 (54.4)	0.73 (0.42)	213 (55.6)	156 (57.6)	0.25 (0.63)	199 (56.9)	170 (55.9)	0.06 (0.81)	227 (56.5)	142 (56.3)	0.00 (1.00)
	Over 1/week	162 (42.2)	123 (45.6)		170 (44.4)	115 (42.2)		151 (43.1)	134 (44.1)		175 (43.5)	110 (43.7)	
Sexual experience	Never experienced	143 (37.2)	53 (19.6)	23.42 (<0.001)	106 (27.7)	90 (22.2)	2.32 (0.14)	101 (28.9)	95 (31.2)	0.44 (0.55)	108 (26.9)	88 (34.9)	4.79 (0.04)
	Experienced	241 (62.8)	217 (80.4)		277 (72.3)	181 (66.8)		249 (71.1)	209 (68.8)		294 (73.1)	164 (65.1)	
Numbers of sexual partner (*n =* 458)	One	84 (34.9)	88 (40.6)	1.58 (0.21)	106 (38.3)	66 (36.5)	0.15 (0.77)	107 (43.0)	65 (31.1)	6.83 (0.01)	120 (40.8)	52 (31.7)	3.73 (0.06)
	Multiple	157 (65.1)	129 (59.4)		171 (61.7)	115 (63.5)		142 (57.0)	144 (68.9)		174 (59.2)	112 (68.3)	
Previous STI (*n =* 458)	No	231 (95.9)	214 (98.6)	3.17 (0.09)	269 (97.1)	176 (97.2)	0.01 (1.00)	244 (98.0)	201 (96.2)	1.36 (0.27)	288 (98.0)	157 (95.7)	1.89 (0.24)
	Yes	10 (4.1)	3 (1.4)		8 (2.9)	5 (2.8)		5 (2.0)	8 (3.8)		6 (2.0)	7 (4.3)	
Previous ECP use	No	341 (88.8)	239 (88.5)	0.01 (0.90)	343 (89.6)	237 (87.5)	0.70 (0.45)	315 (90.0)	265 (87.2)	1.30 (0.27)	360 (89.6)	220 (87.3)	0.78 (0.38)
	Yes	43 (11.2)	31 (11.5)		40 (10.4)	34 (12.5)		35 (10.0)	39 (12.8)		42 (10.4)	32 (12.7)	
Previous condom use	No	153 (39.8)	54 (20.0)	28.86 (<0.001)	113 (29.5)	94 (34.7)	1.97 (0.17)	104 (29.7)	103 (33.9)	1.31 (0.27)	112 (27.9)	95 (37.7)	6.93 (0.01)
	Yes	231 (60.2)	216 (80.0)		270 (70.5)	177 (65.3)		246 (70.3)	201 (66.1)		290 (72.1)	157 (62.3)	
Previous use of oral contraceptive pills	No	343 (89.3)	238 (88.1)	0.22 (0.71)	357 (93.2)	224 (82.7)	17.83 (<0.001)	298 (85.1)	283 (93.1)	10.37 (<0.001)	347 (86.3)	234 (92.9)	6.68 (0.01)
	Yes	41 (10.7)	32 (11.9)		26 (6.8)	47 (17.3)		52 (14.9)	21 (6.9)		55 (13.7)	18 (7.1)	
Previous use of withdrawal method	No	304 (79.2)	222 (82.2)	0.94 (0.37)	313 (81.7)	213 (78.6)	0.99 (0.37)	316 (90.3)	210 (69.1)	46.48 (<0.001)	337 (83.8)	189 (75.0)	7.67 (0.01)
	Yes	80 (20.8)	48 (17.8)		70 (18.3)	58 (21.4)		34 (9.7)	94 (30.9)		65 (16.2)	63 (25.0)	
Previous use of rhythm method	No	281 (73.2)	196 (72.6)	0.03 (0.93)	278 (72.6)	199 (73.4)	0.06 (0.86)	266 (76.0)	211 (69.4)	3.58 (0.06)	324 (80.6)	153 (60.7)	31.02 (<0.001)
	Yes	103 (26.8)	74 (27.4)		105 (27.4)	72 (26.6)		84 (24.0)	93 (30.6)		78 (19.4)	99 (39.3)	
Previous unwanted sex (*n =* 458)	No	221 (91.7)	205 (94.5)	0.22 (0.71)	265 (95.7)	161 (89.0)	7.60 (0.01)	237 (95.2)	189 (90.4)	3.95 (0.06)	276 (93.9)	150 (91.5)	0.94 (0.34)
	Yes	20 (8.3)	12 (5.5)		12 (4.3)	20 (11.0)		12 (4.8)	20 (9.6)		18 (6.1)	14 (8.5)	
Previous unwanted pregnancy (*n =* 458)	No	224 (92.9)	210 (96.8)	3.37 (0.09)	264 (95.3)	170 (93.9)	0.42 (0.53)	239 (96.0)	195 (93.3)	1.65 (0.21)	282 (95.9)	152 (92.7)	2.22 (0.19)
	Yes	17 (7.1)	7 (3.2)		13 (4.7)	11 (6.1)		10 (4.0)	14 (6.7)		12 (4.1)	12 (7.3)	
Previous abortion (*n =* 458)	No	229 (95.0)	216 (99.5)	8.45 (<0.01)	271 (97.8)	174 (96.1)	1.15 (0.39)	245 (98.4)	200 (95.7)	3.00 (0.10)	288 (98.0)	157 (95.7)	1.89 (0.24)
	Yes	12 (5.0)	1 (0.5)		6 (2.2)	7 (3.9)		4 (1.6)	9 (4.3)		6 (2.0)	7 (4.3)	
ECP use is necessary	Disagree	8 (2.1)	7 (2.6)	0.18 (0.79)	12 (3.1)	3 (1.1)	2.91 (0.11)	6 (1.7)	9 (3.0)	1.13 (0.31)	10 (2.5)	5 (2.0)	0.18 (0.79)
	Agree	376 (97.9)	263 (97.4)		371 (96.9)	268 (98.9)		344 (98.3)	295 (97.0)		392 (97.5)	247 (98.0)	
ECP should be available OTC	Disagree	158 (41.1)	96 (35.6)	2.09 (0.17)	154 (40.2)	100 (36.9)	0.73 (0.42)	123 (35.1)	131 (43.1)	4.33 (0.04)	150 (37.3)	104 (41.3)	1.02 (0.32)
	Agree	226 (58.9)	174 (64.4)		229 (59.8)	171 (63.1)		227 (64.9)	173 (56.9)		252 (62.7)	148 (58.7)	
ECP should be prescribed by a doctor	Disagree	244 (63.5)	170 (63.0)	0.02 (0.93)	230 (60.1)	184 (67.9)	4.20 (0.05)	216 (61.7)	198 (65.1)	0.82 (0.37)	250 (62.2)	164 (65.1)	0.56 (0.51)
	Agree	140 (36.5)	100 (37.0)		153 (39.9)	87 (32.1)		134 (38.3)	106 (34.9)		152 (37.8)	88 (34.9)	
ECP is necessary for women’s health	Disagree	93 (24.2)	53 (19.6)	1.93 (0.18)	86 (22.5)	60 (22.1)	0.01 (1.00)	72 (20.6)	74 (24.3)	1.33 (0.26)	80 (19.9)	66 (26.2)	3.53 (0.07)
	Agree	291 (75.8)	217 (80.4)		297 (77.5)	211 (77.9)		278 (79.4)	230 (75.7)		322 (80.1)	186 (73.8)	
Rape	Disagree	2 (0.5)	0 (0.0)	1.41 (0.51)	1 (0.3)	1 (0.4)	0.06 (1.00)	1 (0.3)	1 (0.3)	0.01 (1.00)	0 (0.0)	2 (0.8)	3.20 (0.15)
	Agree	382 (99.5)	270 (100.0)		382 (99.7)	270 (99.6)		349 (99.7)	303 (99.7)		402 (100.0)	250 (99.2)	
Condom breakage	Disagree	67 (17.4)	21 (7.8)	12.73 (<0.001)	59 (15.4)	29 (10.7)	3.02 (0.10)	37 (10.6)	51 (16.8)	5.38 (0.02)	49 (12.2)	39 (15.5)	1.44 (0.24)
	Agree	317 (82.6)	249 (92.2)		324 (84.6)	242 (89.3)		313 (89.4)	253 (83.2)		353 (87.8)	213 (84.5)	
Unwanted sex	Disagree	39 (10.2)	23 (8.5)	0.50 (0.50)	43 (11.2)	19 (7.0)	3.29 (0.08)	28 (8.0)	34 (11.2)	1.92 (0.18)	36 (9.0)	26 (10.3)	0.34 (0.59)
	Agree	345 (89.8)	247 (91.5)		340 (88.8)	252 (93.0)		322 (92.0)	270 (88.8)		366 (91.0)	226 (89.7)	
ECP reduce unwanted pregnancy	Disagree	41 (10.7)	18 (6.7)	3.11 (0.10)	35 (9.1)	24 (8.9)	0.02 (1.00)	25 (7.1)	34 (11.2)	3.24 (0.15)	34 (8.5)	25 (9.9)	0.40 (0.58)
	Agree	343 (89.3)	252 (93.3)		348 (90.9)	247 (91.1)		325 (92.9)	270 (88.8)		368 (91.5)	227 (90.1)	
ECP can cause sex with multiple partners	Disagree	267 (69.5)	191 (70.7)	0.11 (0.80)	263 (68.7)	195 (72.0)	0.82 (0.39)	254 (72.6)	204 (67.1)	2.32 (0.15)	296 (73.6)	162 (64.3)	6.45 (0.01)
	Agree	117 (30.5)	79 (29.3)		120 (31.3)	76 (28.0)		96 (27.4)	100 (32.9)		106 (26.4)	90 (35.7)	
Maximum time to take ECP	Incorrect	257 (66.9)	177 (65.6)	0.13 (0.74)	261 (68.1)	173 (63.8)	1.32 (0.28)	225 (64.3)	209 (68.8)	1.45 (0.25)	264 (65.7)	170 (67.5)	0.22 (0.67)
	Correct	127 (33.1)	93 (34.4)		122 (31.9)	98 (36.2)		125 (35.7)	95 (31.2)		138 (34.3)	82 (32.5)	
Women (*n =* 556)													
Age	18–22	213 (60.5)	132 (64.7)	0.97 (0.37)	187 (59.7)	158 (65.0)	1.62 (0.22)	170 (63.7)	175 (60.6)	0.57 (0.48)	199 (61.8)	146 (62.4)	0.02 (0.93)
	23–41	139 (39.5)	72 (35.3)		126 (40.3)	85 (35.0)		97 (36.3)	114 (39.4)		123 (38.2)	88 (37.6)	
Religion	No	153 (43.5)	71 (34.8)	4.03 (0.05)	132 (42.2)	92 (37.9)	1.06 (0.34)	97 (36.3)	127 (43.9)	3.35 (0.07)	121 (37.6)	103 (44.0)	2.34 (0.14)
	Yes	199 (56.5)	133 (65.2)		181 (57.8)	151 (62.1)		170 (63.7)	162 (56.1)		201 (62.4)	131 (56.0)	
Smoking	Nonsmoking	335 (95.2)	193 (94.6)	0.09 (0.84)	301 (96.2)	227 (93.4)	2.16 (0.17)	251 (94.0)	277 (95.8)	0.98 (0.34)	301 (93.5)	227 (97.0)	3.53 (0.08)
	smoking	17 (4.8)	11 (5.4)		12 (3.8)	16 (6.6)		16 (6.0)	12 (4.2)		21 (6.5)	7 (3.0)	
Alcohol drinking	Rare	253 (71.9)	142 (69.6)	0.32 (0.63)	227 (72.5)	168 (69.1)	0.76 (0.40)	185 (69.3)	210 (72.7)	0.77 (0.40)	223 (69.3)	172 (73.5)	1.19 (0.30)
	Over 1/week	99 (28.1)	62 (30.4)		86 (27.5)	75 (30.9)		82 (30.7)	79 (27.3)		99 (30.7)	62 (26.5)	
Sexual experience	Never experienced	156 (44.3)	59 (28.9)	12.91 (<0.001)	119 (38.0)	96 (39.5)	0.13 (0.73)	100 (37.5)	115 (39.8)	0.32 (0.60)	112 (34.8)	103 (44.0)	4.87 (0.03)
	Experienced	196 (55.7)	145 (71.1)		194 (62.0)	147 (60.5)		167 (62.5)	174 (60.2)		210 (65.2)	131 (56.0)	
Numbers of sexual partner (*n =* 341)	One	83 (42.3)	68 (46.9)	0.70 (0.44)	88 (45.4)	63 (42.9)	0.21 (0.66)	79 (47.3)	72 (41.4)	1.21 (0.28)	91 (43.3)	60 (45.8)	0.20 (0.66)
	Multiple	113 (57.7)	77 (53.1)		106 (54.6)	84 (57.1)		88 (52.7)	102 (58.6)		119 (56.7)	71 (54.2)	
Previous STI (*n =* 341)	No	182 (92.9)	138 (95.2)	0.77 (0.50)	182 (93.8)	138 (93.9)	0.00 (1.00)	161 (96.4)	159 (91.4)	3.73 (0.07)	198 (94.3)	122 (93.1)	0.19 (0.65)
	Yes	14 (7.1)	7 (4.8)		12 (6.2)	9 (6.1)		6 (3.6)	15 (8.6)		12 (5.7)	9 (6.9)	
Previous ECP use	No	295 (83.8)	153 (75.0)	6.40 (0.01)	253 (80.8)	195 (80.2)	0.03 (0.91)	219 (82.0)	229 (79.2)	0.69 (0.45)	252 (78.3)	196 (83.8)	2.62 (0.13)
	Yes	57 (16.2)	51 (25.0)		60 (19.2)	48 (19.8)		48 (18.0)	60 (20.8)		70 (21.7)	38 (16.2)	
Previous condom use	No	174 (49.4)	60 (29.4)	21.24 (<0.001)	129 (41.2)	105 (43.2)	0.22 (0.67)	108 (40.4)	126 (43.6)	0.57 (0.49)	115 (35.7)	119 (50.9)	12.75 (<0.001)
	Yes	178 (50.6)	144 (70.6)		184 (58.8)	138 (56.8)		159 (59.6)	163 (56.4)		207 (64.3)	115 (49.1)	
Previous use of oral contraceptive pills	No	289 (82.1)	170 (83.3)	0.14 (0.73)	291 (93.0)	168 (69.1)	53.96 (<0.001)	213 (79.8)	246 (85.1)	2.75 (0.12)	250 (77.6)	209 (89.3)	12.83 (<0.001)
	Yes	63 (17.9)	34 (16.7)		22 (7.0)	75 (30.9)		54 (20.2)	43 (14.9)		72 (22.4)	25 (10.7)	
Previous use of withdrawal method	No	290 (82.4)	167 (81.9)	0.02 (0.91)	249 (79.6)	208 (85.6)	3.41 (0.07)	240 (89.9)	217 (75.1)	20.77 (<0.001)	279 (86.6)	178 (76.1)	10.36 (<0.01)
	Yes	62 (17.6)	37 (18.1)		64 (20.4)	35 (14.4)		27 (10.1)	72 (24.9)		43 (13.4)	56 (23.9)	
Previous use of rhythm method	No	271 (77.0)	137 (67.2)	6.39 (0.01)	220 (70.3)	188 (77.4)	3.51 (0.07)	202 (75.7)	206 (71.3)	1.36 (0.25)	263 (81.7)	145 (62.0)	26.96 (<0.001)
	Yes	81 (23.0)	67 (32.8)		93 (29.7)	55 (22.6)		65 (24.3)	83 (28.7)		59 (18.3)	89 (38.0)	
Previous unwanted sex (*n =* 341)	No	156 (79.6)	120 (82.8)	0.54 (0.49)	165 (85.1)	111 (75.5)	4.94 (0.04)	133 (79.6)	143 (82.2)	0.36 (0.58)	170 (81.0)	106 (80.9)	0.00 (1.00)
	Yes	40 (20.4)	25 (17.2)		29 (14.9)	36 (24.5)		34 (20.4)	31 (17.8)		40 (19.0)	25 (19.1)	
Previous unwanted pregnancy (*n =* 341)	No	180 (91.8)	142 (97.9)	5.88 (0.02)	186 (95.9)	136 (92.5)	1.79 (0.23)	159 (95.2)	163 (93.7)	0.38 (0.64)	199 (94.8)	123 (93.9)	0.12 (0.81)
	Yes	16 (8.2)	3 (2.1)		8 (4.1)	11 (7.5)		8 (4.8)	11 (6.3)		11 (5.2)	8 (6.1)	
Previous abortion (*n =* 341)	No	181 (92.3)	144 (99.3)	9.04 (<0.01)	188 (96.9)	137 (93.2)	2.57 (0.13)	161 (96.4)	164 (94.3)	0.88 (0.45)	200 (95.2)	125 (95.4)	0.01 (1.00)
	Yes	15 (7.7)	1 (0.7)		6 (3.1)	10 (6.8)		6 (3.6)	10 (5.7)		10 (4.8)	6 (4.6)	
ECP use is necessary	Disagree	2 (0.6)	3 (1.5)	1.18 (0.36)	4 (1.3)	1 (0.4)	1.15 (0.39)	2 (0.7)	3 (1.0)	0.13 (1.00)	1 (0.3)	4 (1.7)	2.98 (0.17)
	Agree	350 (99.4)	201 (98.5)		309 (98.7)	242 (99.6)		265 (99.3)	286 (99.0)		321 (99.7)	230 (98.3)	
ECP should be available OTC	Disagree	151 (42.9)	82 (40.2)	0.39 (0.59)	137 (43.8)	96 (39.5)	1.02 (0.34)	110 (41.2)	123 (42.6)	0.11 (0.80)	138 (42.9)	95 (40.6)	0.28 (0.60)
	Agree	201 (57.1)	122 (59.8)		176 (56.2)	147 (60.5)		157 (58.8)	166 (57.4)		184 (57.1)	139 (59.4)	
ECP should be prescribed by a doctor	Disagree	203 (57.7)	116 (56.9)	0.03 (0.86)	172 (55.0)	147 (60.5)	1.72 (0.20)	145 (54.3)	174 (60.2)	1.98 (0.17)	171 (53.1)	148 (63.2)	5.70 (0.02)
	Agree	149 (42.3)	88 (43.1)		141 (45.0)	96 (39.5)		122 (45.7)	115 (39.8)		151 (46.9)	86 (36.8)	
ECP is necessary for women’s health	Disagree	63 (17.9)	35 (17.2)	0.05 (0.91)	55 (17.6)	43 (17.7)	0.00 (1.00)	46 (17.2)	52 (18.0)	0.06 (0.83)	63 (19.6)	35 (15.0)	1.98 (0.18)
	Agree	289 (82.1)	169 (82.8)		258 (82.4)	200 (82.3)		221 (82.8)	237 (82.0)		259 (80.4)	199 (85.0)	
Rape	Disagree	1 (0.3)	1 (0.5)	0.15 (1.00)	2 (0.6)	0 (0.0)	1.56 (0.51)	0 (0.0)	2 (0.7)	1.85 (0.50)	1 (0.3)	1 (0.4)	0.05 (1.00)
	Agree	351 (99.7)	203 (99.5)		311 (99.4)	243 (100.0)		267 (100.0)	287 (99.3)		321 (99.7)	233 (99.6)	
Condom breakage	Disagree	68 (19.3)	23 (11.3)	6.11 (0.02)	60 (19.2)	31 (12.8)	4.11 (0.05)	35 (13.1)	56 (19.4)	3.98 (0.05)	42 (13.0)	49 (20.9)	6.17 (0.02)
	Agree	284 (80.7)	181 (88.7)		253 (80.8)	212 (87.2)		232 (86.9)	233 (80.6)		280 (87.0)	185 (79.1)	
Unwanted sex	Disagree	25 (7.1)	14 (6.9)	0.11 (1.00)	27 (8.6)	12 (4.9)	2.85 (0.10)	17 (6.4)	22 (7.6)	.033 (0.62)	21 (6.5)	18 (7.7)	0.29 (0.62)
	Agree	327 (92.9)	190 (93.1)		286 (91.4)	231 (95.1)		250 (93.6)	267 (92.4)		301 (93.5)	216 (92.3)	
ECP reduce unwanted pregnancy	Disagree	27 (7.7)	13 (6.4)	0.33 (0.61)	22 (7.0)	18 (7.4)	0.03 (0.87)	18 (6.7)	22 (7.6)	17 (5.3)	17 (5.3)	23 (9.8)	4.20 (0.05)
	Agree	325 (92.3)	191 (93.6)		291 (93.0)	225 (92.6)		249 (93.3)	267 (92.4)		305 (94.7)	211 (90.2)	
ECP can cause sexwith multiple partners	Disagree	227 (64.5)	141 (69.1)	1.24 (0.31)	197 (62.9)	171 (70.4)	3.38 (0.07)	181 (67.8)	187 (64.7)	0.59 (0.47)	220 (68.3)	148 (63.2)	1.56 (0.24)
	Agree	125 (35.5)	63 (30.9)		116 (37.1)	72 (29.6)		86 (32.2)	102 (35.3)		102 (31.7)	86 (36.8)	
Maximum time to take ECP time	Incorrect	226 (64.2)	126 (61.8)	0.33 (0.59)	202 (64.5)	150 (61.7)	0.46 (0.54)	160 (59.9)	192 (66.4)	2.53 (0.11)	196 (60.9)	156 (66.7)	1.96 (0.18)
	Correct	126 (35.8)	78 (38.2)		111 (35.5)	93 (38.3)		107 (40.1)	97 (33.6)		126 (39.1)	78 (33.3)	

*STI* sexually transmitted infection; *OTC* over the counter; *ECP* emergency contraceptive pills

The results of an adjusted logistic regression analysis presented in Table 5 reveal that the most significant influence of the belief that ECP awareness of students’ contraceptive intentions was that “ECP can cause sex with multiple sexual partners” was associated with the intention to use the rhythm method among male students (AOR = 1.61, 95 % CI = 1.02–2.56, *p* <0.05). In contrast, “ECP is necessary in case of condom breakage” was associated with intention to use the withdrawal (AOR = 0.58, 95 % CI = 0.37–0.93, *p* <0.05) and rhythm methods (AOR = 0.36, 95 % CI = 0.16–0.84, *p* <0.05), and “ECP should be prescribed by a doctor” was associated with intention to use the rhythm method (AOR = 0.45, 95 % CI = 0.26–0.77, *p* <0.01) in the female students.

**Table 5:** Factors influencing four contraceptive intentions by sex

	Intention to use condom	Intention to use oral contraceptive pills	Intention to use withdrawal method	Intention to use rhythm method
Men (*n =* 654)				
Demographic and sexual history characteristics				
Age (ref, 18–23 years)	0.82 (0.56–1.20)	–	–	–
Religion (ref, no)	–	–	–	0.66 (0.42–1.03)
Numbers of sexual partner (ref, < 3)	–	–	1.52 (1.00–2.32)	1.41 (0.90–2.19)
Previous condom use (ref, no)	9.99 (1.26–79.12)*	–	–	0.25 (0.06–1.01)
Previous use of oral contraceptive pills (ref, no)	–	3.53 (2.08–6.00)***	0.36 (0.20–0.66)**	0.42 (0.23–0.79)**
Previous use of withdrawal method (ref, no)	–	–	4.99 (3.12–7.95)***	1.48 (0.93–2.36)
Previous use of rhythm method (ref, no)	–	–	–	4.18 (2.70–6.46)***
Previous unwanted sex (ref, no)	–	2.58 (1.21–5.51)*	–	–
Previous abortion (ref, no)	0.09 (0.01–0.67)*	–	–	–
ECP awareness (ref, disagree)				
ECP should be available at OTC	–	–	0.83 (0.54–1.29)	–
ECP should be prescribed by a doctor	–	0.67 (0.44–1.01)	–	–
ECP is necessary for condom breakage	1.54 (0.78–3.05)	–	0.56 (0.26–1.18)	
ECP can cause sex with multiple partners	–	–	–	1.61 (1.02–2.56)*
Women (*n =* 556)				
Demographic and sexual history characteristics				
Religion (ref, no)	1.39 (0.86–2.23)	–	–	–
Numbers of sexual partner (ref, < 2)	0.74 (0.46–1.18)	–	–	0.85 (0.50–1.45)
Previous ECP use (ref, no)	1.36 (0.83–2.25)	–	–	–
Previous condom use (ref, no)	12.60 (1.62–97.99)*	–	–	0.12 (0.03–0.49)**
Previous use of oral contraceptive pills (ref, no)	–	7.89 (4.54–13.70)***	–	0.57 (0.31–1.04)
Previous use of withdrawal method (ref, no)	–	–	3.05 (1.89–4.94)***	2.20 (1.25–3.86)**
Previous use of rhythm method (ref, no)	1.37 (0.87–2.17)	–	–	5.78 (3.43–9.75)***
Previous unwanted pregnancy (ref, no)	1.58 (0.20–12.46)	1.62 (0.57–4.63)	–	–
Previous abortion (ref, no)	0.06 (0.00–1.12)	–	–	–
ECP awareness (ref, disagree)				
ECP should be prescribed by a doctor	–	–	–	0.45 (0.26–0.77)**
ECP is necessary for condom breakage	2.25 (0.95–5.32)	1.37 (0.61–3.11)	0.58 (0.37–0.93)*	0.36 (0.16–0.84)*
ECP will reduce unwanted pregnancy	–	–	–	1.23 (0.39–3.85)

*OTC* over the counter; *ECP* emergency contraceptive pills

*p < 0.05, **p < 0.01, ***p < 0.001

The possible influences of the demographic and sexual history characteristics on contraceptive intentions are as follows. The factors that were significantly related to the intention to use condoms were previous condom use (AOR = 9.99, 95 % CI = 1.26–79.12, *p* <0.05) and previous abortion experience (AOR = 0.99, 95 % CI = 0.01–0.67, *p* <0.05) in the male students, and previous condom use (AOR = 12.60, 95 % CI = 1.62–97.99, *p* <0.05) in the female students. In regard to the intention to use oral contraceptive pills, the significant factors were previous use of oral contraceptive pills (AOR = 3.53, 95 % CI = 2.08–6.00, *p* <0.001) and previous unwanted sex (AOR = 2.58, 95 % CI = 1.21–5.51, *p* <0.05) in the male students, whereas the significant factor in the female students was previous use of oral contraceptive pills (AOR = 3.53, 95 % CI = 2.08–6.00, *p* <0.001). In regard to the intention to use the withdrawal method, the significant factors were previous use of oral contraceptive pills (AOR = 0.36, 95 % CI = 0.20–0.66, *p* <0.01) and previous use of the withdrawal method (AOR = 4.99, 95 % CI = 3.12–7.95, *p* <0.001) in the male students, whereas the significant factor in the female students was previous use of the withdrawal method (AOR = 3.05, 95 % CI = 1.89–4.94, *p* <0.001). Finally, in regard to the intention to use the rhythm method, the significant factors were previous use of oral contraceptive pills (AOR = 0.42, 95 % CI = 0.23–0.79, *p* <0.01) and the previous use of the rhythm method (AOR = 4.18, 95 % CI = 2.70–6.46, *p* <0.001) in the male students, whereas the significant factors in the female students were previous condom use (AOR = 0.12, 95 % CI = 0.03–0.49, *p* <0.01), previous use of the withdrawal method (AOR = 2.20, 95 % CI = 1.25–3.86, *p* <0.01), and previous use of the rhythm method (AOR = 5.78, 95 % CI = 3.43–9.75, *p* <0.001).

## Discussion

This study confirmed sex differences in some areas of ECP awareness and contraceptive intentions and the association and the possible influences of ECP awareness on contraceptive intentions between male and female university students in Korea. Our sex-specific assessment was relevant in comprehending the contraceptive intentions of both sexes. Using our study results, the role of ECP awareness could be expanded into the prediction of other contraceptive intentions in university students.

The majority (88.2 %) of students were aware of the use of ECP. Women were more receptive of the use of ECP in this study, which is similar to the findings in other countries [4, 19]. However, concerning the possible maximum window of time for taking ECP, almost 90 % of students answered earlier than 72 h after unwanted sex, with a low accurate response within 72 h for that question (35 %). However, if students misperceive the time for taking ECP as being shorter, the opportunity for taking an ECP might be abandoned at a time when its use is critical. ECP has been available in Korea for over 10 years. The study results indicate that the provision of accurate information about ECP is still necessary, in particular, to support ECP education in the university setting [2, 4, 5, 12]. In general, students shared favourable opinions about ECP. More than half of the students (56.8 %) responded that they agreed that ECP should be available as an over-the-counter drug and only 39.4 % of students agreed that ECP should be prescribed by a doctor, which implies that many students would like to get ECP when necessary without a doctor visit, which is consistent with previous findings [4, 20].

The ECP policy related to doctors’ prescriptions in Korea has caused conflicts or debates since 2001 among many interested parties such as religious groups, doctors, pharmacists, social activists, and ECP users [21]. Until now, doctors’ opinions have prevailed in Korea, which maintains that counselling or discussion of anything ECP-related should be performed by a doctor to maintain privacy than by a pharmacist, which would expose ECP users to the public [21, 22]. The young population should receive ECP education with sufficient and qualitative information regardless of the Korean ECP policy, which could spread across the nation.

Surprisingly, one study found that ECP could be perceived in a continuum from contraception to abortion, in which 19 % of university students in the US thought that ECP was perceived as an abortive method more than a contraceptive method [5]. In this study, 21.2 % of students had used ECP previously, which is an increase from the data reported 6 years previously in Korea (13.2 %) [2], but it is less than that in Western countries (35–65 %) [3, 7]. The noticeable finding from the current study was that those who used ECP before had greater approval for using ECP for women’s health were male students, and this finding did not appear in the female students. Further study will identify the reasons for the positive change in attitude about ECP-related women’s health.

Meaningful sex differences were found in the results for ECP awareness associated with contraceptive intentions, which provided tips for contraceptive education. Regarding ECP awareness related to sex with multiple partners, male students agreed that they were prone to choose a relatively natural method (such as the rhythm method in this study). However, this method has higher failure, is the least effective of the contraceptive methods, and men themselves do not take responsibility during sex. Based on this finding, ECP awareness related to sex with multiple partners of male students should be changed by ECP education, and then in turn, male students’ intention to use less-effective methods could be reduced. In contrast, women’s agreement with when ECP is necessary was positively associated with a lower intention to use ineffective methods (the withdrawal and rhythm methods in this study). This means information about ECP use especially for emergency situations will be promoted to female students to reduce their intention to use ineffective methods. Another interesting finding was that women’s agreement with prescription of ECP was negatively associated with the intention to use the rhythm method. Female students appeared to have a more conservative attitude toward obtaining ECP; therefore, it is expected that they would carefully prepare or plan to use an effective contraceptive. The role of sexual history characteristics in association with contraceptive intentions, in particular, previously used contraceptives, were all predictors of the intention to use each method regardless of sex, and this seems to be natural.

The most distinctive feature of this study was that the multiple assessment of four kinds of contraceptive intentions were made at a time that differs from previous studies. Accordingly, the comparisons of the current study’s findings to other findings could be given consideration. For example, in condom use among the related studies, the ranges of condom use by university students were differently reported as being from 18.7 to 73.6 % [1, 14]. This may be because of the different measurement methods applied, such as consistency or correctness in condom use [17]. The strength of this study is the confirmation of the association and the possible influences of ECP awareness on intentions to use other contraceptives with a sex-specific approach. Despite the recruitment of subjects from one university, participants seemed to be representative of their university. ECP education should be provided to young people of both sexes, with mandatory counselling and follow-up, particularly for students before and after taking ECP prescribed by the university health centre. If university students were unmarried and they needed contraceptives, then their concerns should be satisfied regardless of the students’ sex. In a recent study, however, students expressed the need for better information about ineffective contraceptive methods than for other methods [12]. Empowering both sexes is necessary to make them aware of ineffective contraception and plan a more effective or reliable contraceptive choice in future.

The limitations for this study include that the intention to use ECP was not considered as a dependent variable because ECP was assumed to be used as a backup, unplanned method. Therefore, the relationship between ECP awareness and the intention to use ECP was not examined.

Researchers should take care when attempting to generalize the study results from one university to other students. Unfortunately, partners’ intentions or decision-making relationships were not measured in this study. However, asking each student to imagine having a discussion with their partner provided the momentum to recognize the importance of communication during sexual encounters about contraceptive behaviour. This study applied a Web-based method to maintain privacy for the students’ responses about sexuality, but it might be possible that unmarried university students in this study could underreport the sex-related responses as suggested in the previous study [14].

The effects of health behaviour, including smoking or alcohol drinking, on students’ contraceptive intentions did not appear significant in this study, but future studies should re-evaluate this factor. Contraceptive use in young Korean students was associated with social norms and cultural beliefs [14]; therefore, it is necessary to compare the cultural influence on the contraceptive intentions of this study with those of other countries.

## Conclusions

The study results confirmed sex differences in ECP awareness in association with contraceptive intentions among university students. Therefore, sex-specific assessments of contraceptive intentions and their associated factors in unmarried university students are necessary. Enhancing positive ECP awareness in both sexes of university students in Korea would be helpful to reduce their intentions to use ineffective contraceptive methods.

## Abbreviations

ECP: Emergency contraceptive pills
STI: Sexually transmitted infection
OTC: Over the counter
